# Occurrence and multidrug resistance of *Campylobacter* spp. at duck farms and associated environmental and anthropogenic risk factors in Bangladesh

**DOI:** 10.1186/s12879-021-06834-w

**Published:** 2021-11-07

**Authors:** Md. Nasir Uddin, Sucharit Basu Neogi, Sk Shaheenur Islam, Jannatul Ferdous, Md. Shahidur Rahman Khan, Shinji Yamasaki, S. M. Lutful Kabir

**Affiliations:** 1 Department of Microbiology and Hygiene, Bangladesh Agricultural University, Mymensingh, 2202 Bangladesh; 2 Graduate School of Life and Environmental Sciences, Osaka Prefecture University, Osaka, 598-8531 Japan; 3 Department of Pharmacology, Bangladesh Agricultural University, Mymensingh, 2202 Bangladesh

**Keywords:** *Campylobacter*, Semi-scavenging duck, Risk factors, Multidrug resistance, Bangladesh

## Abstract

**Background:**

The alarming rise in multi-drug resistant (MDR) zoonotic pathogens, including *Campylobacter* spp., has been threatening the health sector globally. In Bangladesh, despite rapid growth in poultry sector little is known about the potential risks of zoonotic pathogens in homestead duck flocks. The aim of this study was to understand the occurrence, species diversity, and multi-drug resistance in *Campylobacter* spp., and identify the associated risk factors in duck farms in Bangladesh.

**Methods:**

The study involved 20 duck farms at 6 sub-districts of Mymensingh, Bangladesh. Monthly occurrence of *Campylobacter* spp. in potential sources at the farms during February-September, 2018, was detected by culture and PCR-based methods. *Campylobacter* isolates were examined for resistance to different antimicrobials. Risk factors, concerning climatic and environmental disposition, farm management, and anthropogenic practices, of *Campylobacter* infection were estimated by participatory epidemiological tools.

**Results:**

Occurrence of *Campylobacter* spp. was detected in overall 36.90% (155/420) samples, more frequently in drinking water (60%, 30/50), followed by cloacal swab (37.50%, 75/200), egg surface swab (35%, 35/100) and soil of the duck resting places (30%, 15/50) but was not detected in feed samples (n = 20). PCR assays distinguished the majority (61.30%, 95/155) of the isolates as *C. coli*, while the rest (38.70%, 60/155) were *C. jejuni*. Notably, 41.7% (25/60) and 31.6% (30/95) strains of *C. jejuni* and *C. coli*, respectively, were observed to be MDR. The dynamics of *Campylobacter* spp., distinctly showing higher abundance during summer and late-monsoon, correlated significantly with temperature, humidity, and rainfall, while sunshine hours had a negative influence. Anthropogenic management-related factors, including, inadequate hygiene practices, use of untreated river water, wet duck shed, flock age (1–6 months), and unscrupulous use of antimicrobials were identified to enhance the risk of MDR *Campylobacter* infection.

**Conclusion:**

The present study clearly demonstrates that duck farms contribute to the enhanced occurrence and spread of potentially pathogenic and MDR *C. coli* and *C. jejuni* strains and the bacterial dynamics are governed by a combined interaction of environmental and anthropogenic factors. A long-term holistic research at the environment-animal-human interface would be integral to divulge health risk reduction approaches tackling the spread of *Campylobacter* spp. from duck farms.

**Supplementary Information:**

The online version contains supplementary material available at 10.1186/s12879-021-06834-w.

## Background

Along with the huge growth of the human population, and consequently, overexploitation of natural resources, habitat degradation, biodiversity loss, and ecosystem disruption, there have been increasing incidences of zoonotic diseases throughout the world. In Bangladesh, one of the most densely populated countries with rapidly growing poultry sector, ducks are in the second position after chicken to meet people’s demand for meat and eggs [1]. Duck farming, as a part of backyard poultry is a significant component of alternative livelihood for the poor communities in this country, and usually the reared ducks are allowed to utilize the semi-scavenging system with less care of veterinary attention [1, 2]. However, ducks are considered as a potential reservoir of zoonotic infectious organisms, including virus species like the highly pathogenic avian influenza virus (HPAIV) and pathogenic bacteria like *Campylobacter* spp. [3, 4]. *Campylobacter* spp. are Gram negative, spiral or slightly curved, microaerophilic, non-spore forming, and motile bacteria with single flagellum at one or both poles [5]. In both developing and developed countries, *Campylobacters* are frequently isolated among the important enteric pathogens associated with gastroenteritis in human populations, especially in children under 5 years, adolescents aged 15–25 years, and immunocompromised persons [6–8]. In the case of campylobacteriosis in humans, *C. jejuni* constitutes an overwhelming majority (> 90%) of the infections, while *C. coli* dominates among the rest cases [7]. In Europe, over 200,000 cases of campylobacteriosis have been reported annually, however, the actual number of cases is estimated to be approximately 9 million [8]. *Campylobacter* spp. have been occasionally reported to inflict Guillain-Barré syndrome, Reactive Arthritis, Miller Fisher syndrome, and Irritable Bowel syndrome [5]. In broiler chicken and cattle, *C. jejuni* is the dominant species, accounting > 80% among the isolates of thermophilic *Campylobacter* spp., whereas *C. coli* predominates (> 90%) among those from wild ducks and swine [3, 7]. In comparison to extensive research on *Campylobacter* spp. among the broiler and layer chickens, there have been relatively less investigations on the occurrence and risk factors of this pathogen among the duck flocks, particularly, in developing countries.

Transmission of *Campylobacter* spp. in human usually happen through consumption of contaminated meat, water and milk products [9]. Apart from unhygienic handling of poultry animals or their edible products, environmental components contaminated with poultry excreta are important risk factors for *Campylobacter* infections, particularly, at farms and abattoirs [10, 11]. A summer peak in the prevalence of *Campylobacter*-positive poultry flocks coinciding with increased infection in humans has been reported in a number of countries [12, 13]. Variation in temperature, 2–6 weeks prior to the reported infections has been notified as to the most important climatic predictor of campylobacteriosis incidence, which may be also influenced by sunshine hours, and relative humidity [14–16].

In the recent decades, the worldwide rise in multi-drug resistance (MDR) among the microbial pathogens at an alarming frequency have imposed a significant challenge to the health of humans and animals. Large-scale applications of antimicrobials, mostly in an imprudent manner, in medical, agriculture, livestock, and poultry sectors are thought to induce selective pressure favoring the occurrence of MDR pathogens [17, 18]. Although *Campylobacter* infections are mostly self-limiting, antimicrobial agents, including erythromycin, tetracycline, aminoglycosides, and fluoroquinolones are often used, particularly, for immunocompromised patients [18–20]. In Bangladesh, despite increasing efforts by the Government to promote the prudent use of antimicrobials, [21] these agents are still widely applied. In this densely populated country, MDR infections from the emergent poultry sector is a major concern to public health. Consequently, *Campylobacter* isolates showing an increasing frequency of resistance to antimicrobials, including nalidixic acid, tetracycline, fluoroquinolones, and macrolides, have been reported for both in healthy animals and human cases of gastroenteritis and diarrhea [10, 11, 22–24].

In Bangladesh, there remains a lack of systematic information on the occurrence, socio-environmental risk factors, and multi-drug resistance of *Campylobacter* spp. in ducks and wild birds in this country. Various studies have noted that apart from climatic influences, other factors related to farm management, e.g., unhygienic practices, increasing age of birds, flock size, insufficient sunlight or ventilation, and even interface with wild birds could influence the occurrence of *Campylobacter* spp. in poultry flocks [11, 24–26]. Despite its natural link with *Campylobacter*, duck or duck farms as a source of campylobacteriosis in humans has remained unexplored in Bangladesh. This study aimed to understand the occurrence of *Campylobacter* spp., and their drug resistance patterns, in semi-scavenging duck flocks, reared at household premises in this country. Moreover, the sociodemographic traits, farm management practices and climatic factors that may contribute to increased occurrence of *Campylobacter* spp. were also assessed to identify the major socio-environmental drivers and risk factors of these potentially harmful bacteria associated with duck farming.

## Methods

### Study sites

**Fig. 1:**
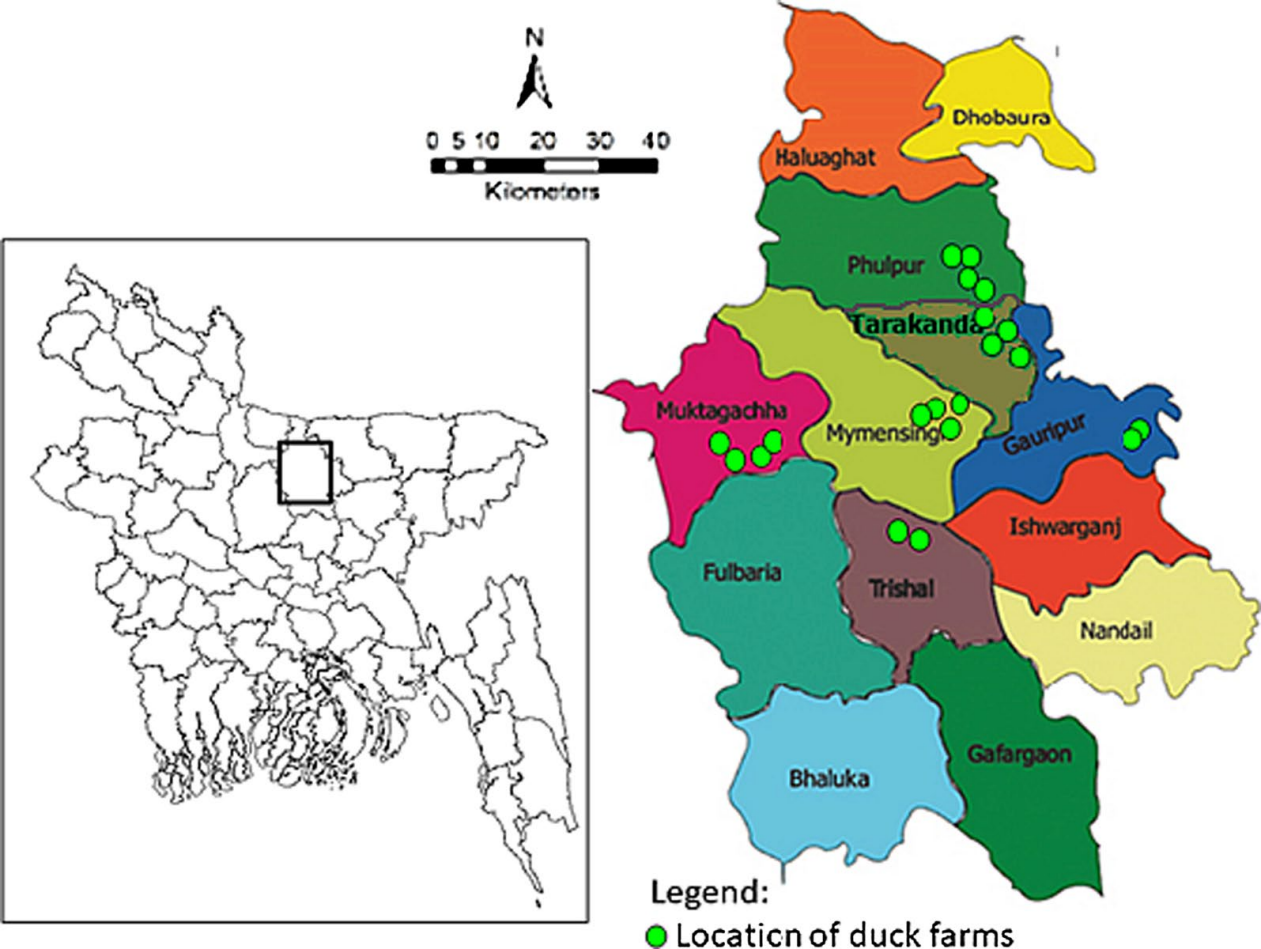
Locations of the duck farms in six sub-districts of Mymensingh district in Bangladesh. A total of 20 duck farms, including 4 from each of Muktagachha, Mymensingh sadar, Tarakanda, and Phulpur sub-districts and 2 from each of Trishal, and Gouripur sub-districts (upazila) were surveyed

The study involved socio-environmental surveillance at 20 semi-scavenging duck farms in 6 sub-districts (Mymensingh Sadar, Muktagachha, Phulpur, Tarakanda, Gouripur and Trisal) of Mymensigh district in Bangladesh (Fig. 1). The study farms were selected based on consultation with local livestock offices, with the inclusion criteria: number of reared ducks at least 200, and a single farm from each village. At least 2 farms from each sub-districts were selected.

### Collection of animal and environmental samples

Samples representing the potential sources of *Campylobacter* spp. in duck farms were collected during April-August, 2018 for microbiological analysis. Considering an expected prevalence of *Campylobacter* spp. in 50% of the ducks, a sample size of 385 at least was needed for a 95% confidence interval, according to the following formula [27].

$$n={Z}^{2}p(1-p)/{d}^{2}$$

where n represents the sample size needed; Z^2^, the Z-score at 95% confidence interval (1.96); p, the expected proportion (50%) of Campylobacter spp.; and d, the expected margin of error (0.05).

However, in this cross-sectional study, five kinds of samples at various proportions were collected at each of the 20 duck farms, yielding a total of 420 samples. The sampling strategy for different contamination sources was in accordance to the observed trend of *Campylobacter* occurrence at poultry farms in this region [11]. The sample diversity represented potential contamination sources: cloacal swab (CS), egg surface swabs (ES), the soil of the duck resting places (S), drinking water (DW), and feed (F). At individual farm level, category-wise sample numbers were CS (n = 10), ES (n = 5), S (n = 2–4), DW (n = 2–4) and F (n = 1), respectively. Overall, from all the duck farms, a total of 200, 100, 50, 50, and 20 samples of CS, ES, S, DW, and F, respectively, were obtained.

During each sampling of DW, S, and F, at least three sub-samples were randomly collected and pooled together. The source of DW was either tube-well or natural surface water of the river/canal. Feed samples represented the commercially formulated products (a mixture of ingredients: cereal grains and plant or animal by-products, vitamin and mineral supplements). These commercial feeds were regularly served to the ducks, which were also allowed for natural scavenging. The ES samples were obtained from the newly laid eggs. Aseptic measures were followed while collecting these samples in various amounts, i.e., 3–5 g (wet weight) for CS and ES, 100 g for soil and feed samples, and 500 mL for DW. The swab samples were collected in a sterile cotton swabs and preserved immediately in Cary-Blair transport media while the rest of the samples were kept in sterile plastic containers. All the samples were transported in an insulated foam box with a cold chain (temperature, 4–6 °C) and processed within 6 h of collection.

### Isolation and identification of *Campylobacter* spp. by culture based methods

Isolation of *Campylobacter* spp. was carried out following the filtration-based culture method [28]. In brief, the swab samples (1 g each) and soil or feed samples (10 g each) were suspended in 1 and 10 mL, respectively, of sterile phosphate buffer saline (pH 7.4). A 100 µl portion, in three replicates, of the suspensions was spread onto a membrane filter (mixed cellulose ester type, 0.45 µm pore size, 47 mm diameter; Sartorius Stedim Biotech, Germany). In the case of DW, at least three replicates of a 100 mL portion were filtered. After sample inoculation, each of the membrane filters was overlaid on a blood agar medium (blood base agar no. 2; HiMedia, India), supplemented with 5% defibrinated sheep blood, and allowed to stand for 30 min at room temperature. The filter paper was then removed and the blood agar plate was incubated at 37 °C for 48 h within a thin-type jar in microaerophilic condition (5% O_2_, 10% CO_2_, and 85% N_2_) using AnaeroPouch^®^-MicroAero (Mitsubishi Gas Chemical Co., Inc.). Presumptive colonies of *Campylobacter*, which appear grey, flat, and irregularly spreading, were screened from the incubated blood agar medium and sub-cultured on the same medium following the procedures described above. The pure culture of each isolate was then subjected to Gram’s staining and observed under a microscope. *Campylobacter*-like isolates, which appeared Gram-negative and curve- or spiral-shaped cells, were subjected to species-differentiating biochemical assays, including motility test, catalase, oxidase, and hippurate hydrolysis, according to standard procedures [5].

### PCR-based confirmation of *Campylobacter* spp.

DNA templates from presumptive isolates of *Campylobacter* spp., detected by culture-based methods, were prepared following the boiling method [29]. The 16S rRNA gene-based PCR was performed for the identification of the genus *Campylobacter* using primers and thermal conditions according to Samosornsuk et al. [30]. Species-specific detection of *C. jejuni* was done following a previously established hippuricase gene-based (*hipO*) PCR assay [31], with a slight adjustment of thermal condition. The identity of *C. coli* was confirmed following the *cdtC* gene-based multiplex PCR assay [32]. All primers (Macrogen Inc., Korea) and thermal conditions for the PCR assays used in this study are described in Table 1. In each PCR, the reaction mixture (25 µl) was prepared by mixing 12 µl master mixtures (Promega, USA), 1 µl of each forward primer (10 pmol), 1 µl each reverse primer (10 pmol), 3 µl DNA template and rest deionized water. The PCR reactions were carried out using a thermocycler (Astec, Japan). PCR products (5 µl) were analyzed by 2% agarose (Invitrogen, USA) gel electrophoresis, followed by staining of the gel with ethidium bromide (0.5 µg mL^−1^) and de-staining with distilled water, 10 min each. Afterward, the gel images with PCR amplicons were captured using a UV transilluminator (Biometra, Germany).

**Table 1:** List of primers and thermal condition used for molecular identification of *Campylobacter* species

Primer	Sequence (5′-3′)	Target	Amplicon size (bp)	PCR condition (30 cycle)			References
				Denature	Annealing	Extension	
16S9F 16S1540R	GAGTTTGATCCTGGCTC AAGGAGGTGATCCAGCC	16S rRNA	1530	94 °C, 30 s	47 °C, 30 s	72 °C, 90 s	Samosornsuk et al. [21]
HIP400F HIP1134R	GAAGAGGGTTTGGGTGGTG AGCTAGCTTCGCATAATAACTTG	*hipO* gene	735	94 °C, 30 s	55 °C, 30 s	72 °C, 45 s	Linton et al. [22]
Cj-cdtCU1 Cj-CdtCR2	TTTAGCCTTTGCAACTCCTA AAGGGGTAGCAGCTGTTAA	Cj*-cdtC*	524	94 °C, 30 s	53 °C, 30 s	72 °C, 30 s	Asakura et al*.* [23]
Cc-CdtCU1 Cc-CdtCR1	TAGGGATATGCACGCAAAG GCTTAATACAGTTACGATAG	Cc*-cdtC*	313				
CfspCU2 CfspCR1	AAGCATAAGTTTTGCAAACG GTTTGGATTTTCAAATGTTCC	Cf-*cdtC*	397				

Cj:* C. jejuni*; Cc:* C. coli*; Cf:* C. fetus*

### Antimicrobial susceptibility test

All isolates of *C. jejuni* and *C. coli* were tested for their antimicrobial susceptibility by disk diffusion method [24]. Eight commonly used antimicrobial agents at standard doses were used: amoxicillin (AMX) (30 µg), azithromycin (AZM) (30 µg), ciprofloxacin (CIP) (5 µg), erythromycin (ERY) (30 µg), gentamycin (GEN) (10 µg), norfloxacin (NOR) (10 µg), streptomycin (STR) (10 µg), and tetracycline (TET) (30 µg) (HiMedia, India). Initially, cells from freshly grown broth-culture (Mueller–Hinton broth, HiMedia, India) of each *Campylobacter* isolate were harvested by centrifugation and then suspended in sterile normal saline (pH 7.4). The turbidity of each cell suspension was adjusted to a 0.5 McFarland standard. A portion of the cell suspension was uniformly inoculated, using a sterile cotton swab, on the entire surface of Muller Hinton agar (HiMedia, India), supplemented with 5% defibrinated sheep blood, to produce a confluent lawn of bacterial growth. After drying of the inoculum, 4 antimicrobial discs were placed on each agar plate, which was then incubated in the inverted position at 37 °C for 48 h under microaerophilic conditions (5% O_2_, 10% CO_2_, and 85% N_2_). The zone of growth inhibition (diameter) of each antimicrobial agent was evaluated according to the interpretative criteria as described by the Clinical and Laboratory Standards Institute [33]. *E. coli* strain ATCC 25,922 was used as a quality control organism. The results were confirmed by conducting at least two replicates of each disc diffusion experiment.

### Surveillance on sociodemographic status, farm management and climatic factors

Participatory epidemiological (PE) methods [34], including semi-structured interviews, direct observation and focus group discussions (FGDs) were conducted during April-August, 2018 to understand the variations in sociodemographic status and farm management practices that may influence the occurrence of *Campylobacter* spp. in the semi-scavenging duck flocks. During each FGD, the participation of at least three duck farmers, including the owner, for each of the selected farms (n = 20) was ensured. Response to individual queries was attained through consensus among the participants. Prior to each survey, conducted by an experienced team comprised of a couple of veterinarians, the study objectives were explained and verbal consent was obtained from the participants. A total of 20 FGDs, one for each selected farm, were conducted according to the standard procedures [35]. Semi-structured questionnaire-based surveillance was made to understand potential risk factors in the duck farms. In this purpose, farm management, hygiene, and environment-related variables, e.g., number, and age of the reared ducks, scavenging sites, type of feed, source of drinking water, floor condition, sunlight and ventilation facilities, cleaning of the floor and feeder, disinfectants use, hand washing, handling of ducks, veterinary health care facilities, use of antimicrobials and vaccines, duck waste disposal, and wild animal/bird-duck interactions were considered (see Additional file 1). Sociodemographic information, e.g., profession, education, training, and experience in duck rearing, were also collected. Results obtained from PE methods were verified by direct observations and recorded in pre-formatted questionnaires in hard copies.

Among the climatic variables, daily data of temperature (minimum, mean, and maximum values), rainfall, relative humidity, and sunshine hours of the study region (Mymensingh District) for the tenure January–September, 2018, were obtained from the archives of AccuWeather (https://www.accuweather.com/). Climatic data were transformed into monthly average and compared with the concurrent occurrence of *Campylobacter* spp. in different components of the duck farms.

### Data management and statistical analyses

Data were imported into Microsoft Excel 10 (Microsoft Corporation, Redmond, WA, USA) spreadsheet from the hard copies, and cleaned, coded, and checked for the integrity of the data set. Descriptive statistics with proportion percentage and 95% confidence interval (CI) of the data representing variations in socio-environmental conditions and bacterial occurrences were performed using the Epi info 7 software [36].

The occurrence of *Campylobacter* for a particular month were compared with overall average values for the concurrent and preceding months of each climatic factor since the bacterial occurrence in poultry was reported to be influenced by climatic variations at the time lag of 2–5 weeks prior [14–16]. The proportion of the farm samples of each category contaminated with *Campylobacter* spp. was compared. Correlation and linear regression analysis between the occurrence of *Campylobacter* spp. and individual climatic factors were performed using ‘Xact’ (ver. 7.21d, SciLab GmbH, St. Yrieix, France). Significant association (considering p < 0.05) between the prevalence of *Campylobacter* species and any individual factors related to farm management was determined by the Chi-square test using Statistica (ver. 10.0, StatSoft Inc., USA).

The antimicrobial susceptibility profile of the bacterial isolates was evaluated according to their differentiation into three independent groups, i.e., resistant, intermediate, and susceptible. MDR trait was defined as bacterial strains showing resistance against at least one antimicrobial agent in three or more antimicrobial classes [37]. A descriptive comparison of resistant patterns of *Campylobacter* strains with respect to diversity in sources and/or anthropogenic factors was performed using mean/ median and standard deviation, and also in the form of the box plots. Differences in the patterns and/or occurrence of antibiotic resistance in *Campylobacter* spp. (*C. coli* and *C. jejuni*) were calculated by the Paired Samples t-test. A ‘p’ value of < 0.05 was considered significant.

## Result

### Isolation, identification and occurrence of *Campylobacter* spp.

Of 420 samples (N) collected from 20 duck farms *Campylobacter* spp. was detected and isolated from 36.90% (95% CI = 32.27–41.72%) samples by selective culture, followed by 16S rRNA gene-based genus-specific PCR method (see Additional file 2 showing gel images of representative PCR products).

Among different categories of samples from the duck farms, the contamination of *Campylobacter* spp., was detected more frequently in drinking water (60%, 30/50), followed by cloacal swabs (37.50%, 75/200) and egg surface swab (35%, 35/100) (Table 2). Among the soil samples collected from the duck resting places 30% (15/50) were detected positive but all feed samples (n = 20) were found to be negative for *Campylobacter* spp. Depending on sample size, the 95% CI for the positive detection rate was estimated to deviate ca. 7–15%, the higher the sample number the lower the percentage deviation (Table 2).

**Table 2:** Summary of the occurrence of *Campylobacter* species isolated from different type of samples of selected semi-scavenging duck farms

Sample (N)/Species	Positive (n)	Occurrence (95% CI)
Cloacal swab (200)		
* Campylobacte*r spp.	75	37.50 (30.78–44.60)
* C. jejuni*	29	14.50 (9.93–20.16)
* C. coli*	46	23 (17.35–29.47)
Egg surface swab (100)		
* Campylobacter* spp.	35	35 (25.73–45.18)
* C. jejuni*	13	13 (7.10–21.20)
* C. coli*	22	22 (14.33–31.39)
Soil of the duck resting places (50)		
* Campylobacter* spp.	15	30 (17.86–44.60)
* C. jejuni*	6	12 (4.53–24.31)
* C. coli*	9	18 (8.57–31.43)
Water (50)		
* Campylobacter* spp.	30	60 (45.17–73.60)
* C. jejuni*	12	24 (13.06–38.16)
* C. coli*	18	36 (22.91–50.80)
Feed (20)	0	0
Overall (420)		
* Campylobacter* spp.	155	36.9 (32.27–41.72)
* C. jejuni*	60	14.28 (11.08–18)
* C. coli*	95	22.62 (18.70–26.92)

*CI* confidence interval

Species-specific detection by PCR assays could differentiate the isolated strains into two species: *C. coli* and *C. jejuni* in 22.62% (95/420, 95% CI = 18.70–26.92%) and 14.28% (60/420, 95% CI = 11.08–18%) of the total samples, respectively. *Campylobacter* isolates which were positive in the biochemical assay for hippurate hydrolysis also yielded positive in hippuricase (*hipO*) gene-based PCR, confirming their identity as *C. jejuni*. On the other hand, all isolates of *Campylobacter* producing negative results in hippurate hydrolysis test were identified as *C. coli* by *cdtC* gene based multiplex PCR (see Additional file 2). Overall, among the isolated strains (n = 155), an almost two-third portion (n = 95) were identified as *C. coli* while the rest were *C. jejuni*. In drinking water samples (n = 50), 24% (95% CI = 13.06–38.16%) and 36% (95% CI = 22.91–50.80%) were observed to be contaminated with *C. jejuni* and *C. coli*, respectively. The higher prevalence of contamination by *C. coli* than *C. jejuni* was also observed to occur at a similar proportion in all other kinds of samples (Table 2).

### Antimicrobial resistance pattern of *C. jejuni* and *C. coli*

**Fig. 2:**
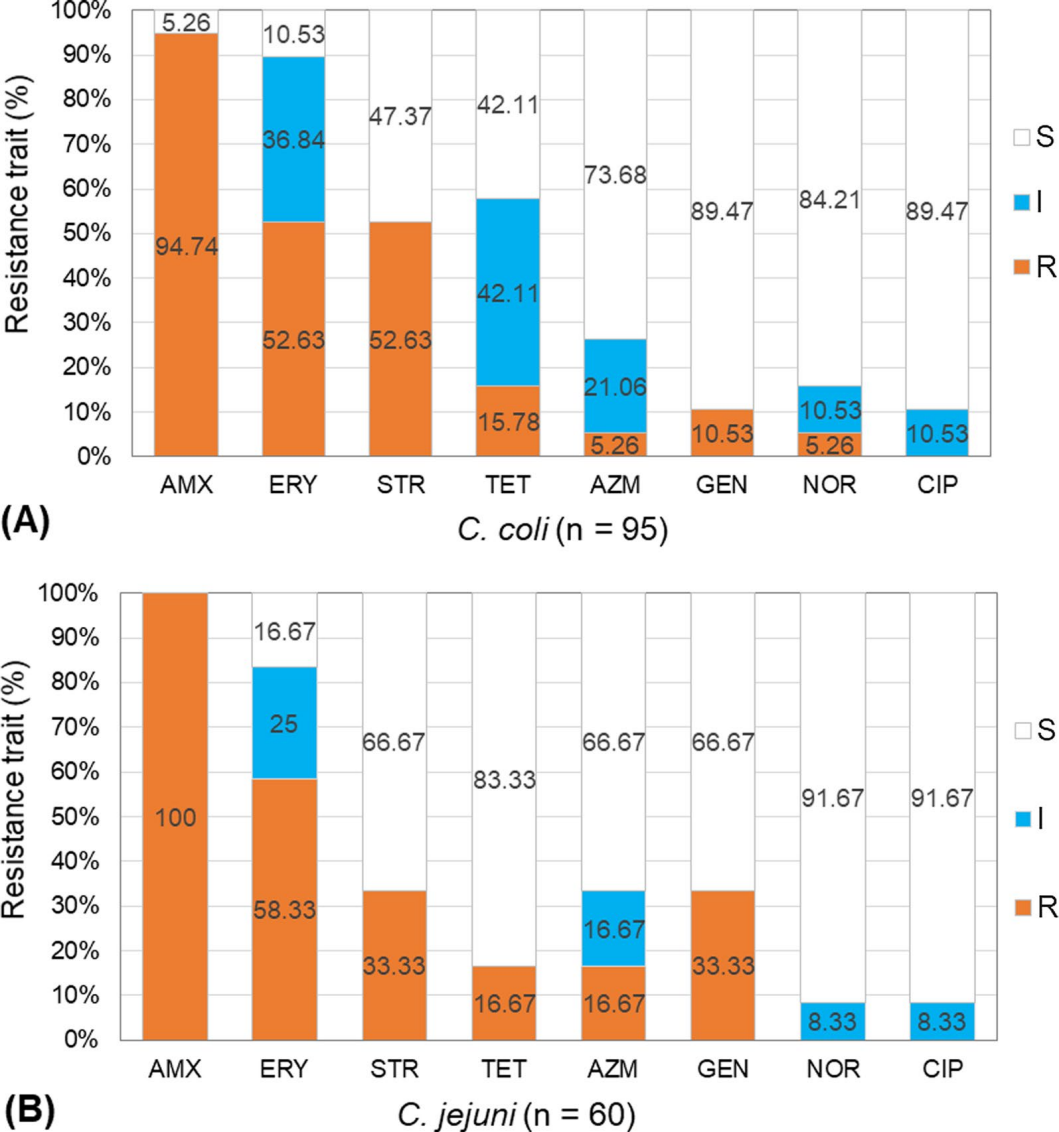
Antimicrobial resistance traits of **A** *C. coli*, and **B** *C. jejuni* strains isolated from duck farms. Eight selected antimicrobial agents were checked at standard doses (μg): Amoxicillin (AMX, 30 µg), Azithromycin (AZM, 30 µg), Ciprofloxacin (CIP, 5 µg), Erythromycin (ERY, 30 µg), Gentamycin (GEN, 10 µg), Norfloxacin (NOR, 10 µg), Streptomycin (STR, 10 µg), and Tetracycline (TET, 30 µg). The resistance traits were categorized into three categories: susceptible (S), intermediate (I), and resistant (R) according to Clinical and Laboratory Standards Institute (CLSI, 2016)

In case of the isolated strains of *C. jejuni* (n = 60), all were observed to be resistant to amoxicillin (Fig. 2). Among these strains, 58.33% (n = 35) showed resistance against erythromycin, while 33.33% (n = 20) were resistant to both streptomycin and gentamicin, and 16.67% (n = 10) to both tetracycline and azithromycin. On the other hand, of 95 strains of *C. coli*, 94.74% (n = 90) exhibited resistant trait for amoxicillin, 52.63% (n = 50) for both erythromycin and streptomycin, and 15.78% (n = 15) for tetracycline (Fig. 2). The observed trend in the percentage occurrence of resistant traits was mostly similar for both the *C. jejuni* and *C. coli* strains. However, a more frequent occurrence of resistance to streptomycin was observed for the *C. jejuni* strains in comparison to *C. coli.* In the case of gentamicin, a higher frequency of resistance for the *C. coli* strains (33.33%) in comparison to *C. jejuni* (10.5%) was notable.

Out of 60 isolated strains of *C. jejuni*, only 8.33% (n = 5) were resistant to one agent (AMX), while 33.33% (n = 20) were resistant to 2 agents in different categories (AMX-GEN, AMX-ERY, AMX-STR, and AMX-TET) (Table 3). Multidrug resistance, i.e., co-resistance to at least three types of antimicrobials were observed for a total of about 42% strains of *C. jejuni*. Among these MDR strains, resistance against 3 antimicrobials, differentiated into two patterns (AMX-AZM-ERY and AMX-ERY-STR) were observed for 16.66% strains of *C. jejuni*, while 25% of them were resistant to 4 agents, differentiated into three patterns (AMX-ERY-STR-TET, AMX-AZM-ERY-GEN and AMX-ERY-GEN-STR). Among the 95 strains of *C. coli*, 15.8% (n = 15) were resistant to one antimicrobial agent (AMX or ERY), whereas almost half portion (47.37%) were resistant to two antimicrobial agents, differentiated into five patterns (AMX-TET, AMX-ERY, AMX-NOR, AMX-STR, and AMX-GEN) (Table 3). Occurrence of MDR strains were observed among a total of 36.84% (n = 35) strains, with resistance to three antimicrobial agents of one pattern (AMX-ERY-STR) and four agents of different combinations (AMX-ERY-STR-TET, AMX-E-GEN-STR, AMX-AZM-ERY-GEN) in 15.8% and 21.05% strains, respectively, of *C. coli*. Overall, multidrug-resistant *Campylobacter* spp., considering those showing resistance to at least three or more antimicrobial classes, were observed to constitute ca. 38.70% (60/155) of the isolated strains. Intermediate resistant isolates, considered as a group independent from those of resistant and susceptible isolates, were observed for erythromycin, tetracycline, azithromycin, norfloxacin, and ciprofloxacin, with a more frequent occurrence in *C. coli* than *C. jejuni* (Fig. 2).

**Table 3:** Antimicrobial resistance profile of *C. jejuni* and *C. coli* isolates from duck farms

*Campylobacter* spp	Category of resistance (against one or multiple antimicrobials)	Resistance patterns^1^	No. (%) of strains	No. (%) of multidrug resistant isolates
*C. jejuni* (n = 60)	Against one	AMX	5 (8.33)	25 (41.67)
	Against two	AMX-GEN	10 (16.67)	
		AMX-ERY	10 (16.67)	
		AMX-STR	5 (8.33)	
		AMX-TET	5 (8.33)	
	Against three	AMX-ERY-STR	5 (8.33)	
		AMX-AZM-ERY	5 (8.33)	
	Against four	AMX-ERY-STR-TET	5 (8.33)	
		AMX-AZM-ERY-GEN	5 (8.33)	
		AMX-ERY-GEN-STR	5 (8.33)	
*C. coli* (n = 95)	Against one	AMX	10 (10.53)	35 (36.84)
		ERY	5 (5.26)	
	Against two	AMX-STR	20 (21.05)	
		AMX-ERY	10 (10.53)	
		AMX-NOR	5 (5.26)	
		AMX-TET	5 (5.26)	
		AMX-GEN	5 (5.26)	
	Against three	AMX-ERY-STR	15 (15.79)	
	Against four	AMX-ERY-STR-TET	10 (10.53)	
		AMX-AZM-ERY-GEN	5 (5.26)	
		AMX-ERY-GEN-STR	5 (5.26)	

^1^Antimicrobial resistance at standard doses (μg): Amoxicillin (AMX, 30 µg), Azithromycin (AZM, 30 µg), Ciprofloxacin (CIP, 5 µg), Erythromycin (ERY, 30 µg), Gentamycin (GEN, 10 µg), Norfloxacin (NOR, 10 µg), Streptomycin (STR, 10 µg), and Tetracycline (TET, 30 µg)

### Climatic variations and *Campylobacter* dynamics in duck farm samples

**Fig. 3:**
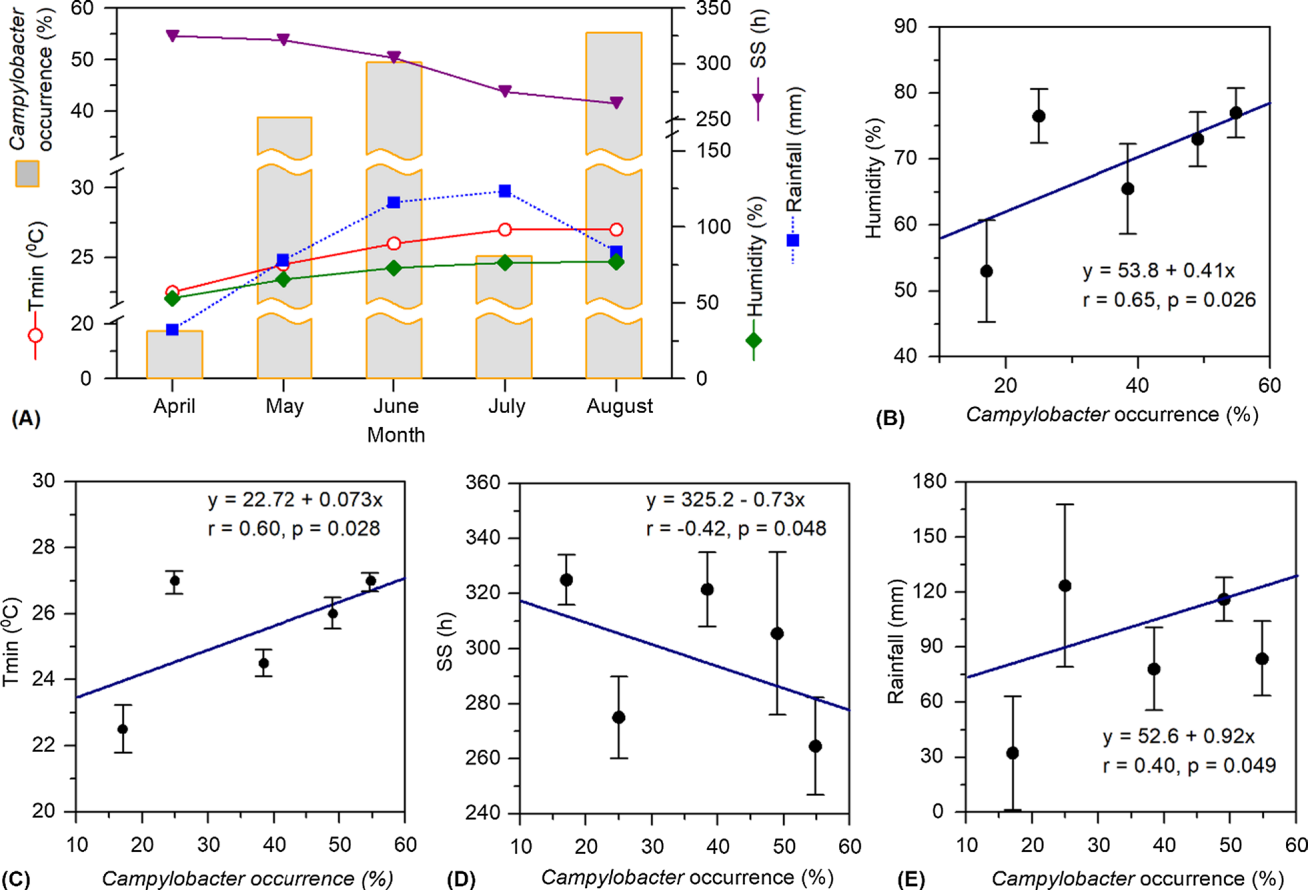
Influence of climatic factors on the occurrence of *Campylobacter* spp. in samples collected from the selected duck farms. **A** Monthly variations in the mean values of the climatic factors and *Campylobacter* abundance in different samples of the duck farms. Each of the mean values was computed as the average of daily values of the month compared and its preceding one. The monthly occurrences of *Campylobacter* spp. represent the bacterial overall isolation rate (% abundance) for different types of samples, i.e., cloacal swab (n = 20–50), egg surface swab (n = 10–25), soil of the duck resting places (n = 5–14) and drinking water (n = 5–14). **B**–**E** Correlations between the occurrence of *Campylobacter* spp. and climatic factors. In each figure, the diagonal line represents linear regression between the two parameters, and the relevant statistical information, including regression equation, r- and p-values are shown for each correlation. Standard deviations of the mean values of each climatic factor are shown as vertical bars. Tmin and SS indicate temperature minimum and sun shine hours, respectively

The monthly occurrence, i.e., overall isolation rate in different samples, of *Campylobacter* spp. in duck farms was observed to be influenced by the variations in temperature, humidity, sunshine hours, and rainfall in the localities. The trend of climatic influence on the bacterial occurrence was observed to be similar among different types of samples with variable isolation rates (see Additional file 3 showing the monthly isolation rates of *Campylobacter* spp. in different samples). The monthly occurrence of *Campylobacter* spp. in duck farm samples showed an increasing trend, ca. 17–49%, accompanied by the rise in temperature from April to June (Fig. 3A). However, *Campylobacter* abundance showed a drastic decrease in July, with an overall isolation rate of 25% in the collected samples. This was likely a consequence of the dilution impact of monsoon-driven intense rainfall during June-July. However, a significant increase in *Campylobacter* occurrence, yielding the highest isolation rate of ca. 55% for overall samples, was observed for the month of August. A large-scale preponderance of *Campylobacter* spp. during this late monsoon period coincided with a warm and humid climate but comparatively low amount of rainfall and less duration of sunshine hours (Fig. 3A). The monthly mean values of temperature minimum (Tmin), obtained from its daily values, represented better the temperature variation in comparison to its maximum (Tmax) and average (Tavg) values. Statistical analysis comparing the bacterial isolation rate with individual climatic factors showed that the dynamics of *Campylobacter* spp. was significantly correlated with Tmin (p = 0.028, r = 0.60) and humidity (p = 0.026, r = 0.65). On the other hand, rainfall and sun shine hours showed a positive and negative influence, respectively, on *Campylobacter* occurrence, however, in both cases, the significance level was comparatively low and the standard deviation of monthly data was relatively high (Fig. 3B–E).

### Duck farm demographics

Of 20 duck farms, a total of 57 representatives who were directly involved in farm management were interviewed. These farm managers included the owners (35.10%, n = 20), his/her spouse (29.82%, n = 17), son (17.54%, n = 10) and hired employees (17.54%, n = 10). Approximately 25% of the duck rearers had no formal education, while ca. 23% of them had completed schooling up to primary level, and the rest had education level between class VI and class XII. Most of the farmers (94.74%, n = 54) had no training on duck farming, especially, farm management, biosecurity, and personal hygiene and sanitation practices. Approximately two-third (n = 37, 64.91%) of the informants had 1 to 5 years of experience in duck rearing, while only a few of them (n = 9, 15.79%) had such experience over 10 years (see Additional file 4 for details).

### Farm management practices and factors influencing *Campylobacter* occurrence

Most of the farms (n = 16, 80%) were rearing the ‘Khaki Kemble’ breed of ducks, while a few (n = 2, 10%) had the ‘Choruy’ breed and rest (n = 2, 10%) of the farms rearing both types. The surveyed farms had variable conditions and practices related to farm management which have been summarized in Additional file 5. The number of reared ducks was small (200–300 ducks) in 40% (n = 8), medium (300–750 ducks) in 30% (n = 6) and large (750–1500 ducks) in 30% (n = 6) of the farms. More than half portion, 55% (n = 11), of the farms had ducks of young ages (1–5 months), whereas the rest other farms (n = 9, 45%) had matured ducks (age 10–15 months) capable of egg production. The sources of drinking water were tube-well water (40%, n = 8) and surface water of river or canal (60%, n = 12). More than two-third portion of the farms (n = 14, 70%) had provision of scavenging feed from watershed and paddy field, however, in other farms, the scavenging sites were the river and paddy field (n = 4, 20%), or pond and paddy field (n = 2, 10%). Access to sunlight was available in 50% of the duck sheds while 20% of the farms reported the interface of ducks’ scavenging area with wild animals/birds. The majority of the farms (n = 17, 85%) used duck manure as fish feed in their ponds. However, all the farms used antimicrobial agents of different kinds, including gentamycin (50%), enrofloxacin (30%) and oxytetracycline (20%), while 60% of the farms applied vaccines against common duck diseases (duck plague and duck cholera).

Concerning hygiene practices, regular cleaning of the feeder and drinker was practiced in 40% (n = 8) of the farms. Among the selected duck farms, floor cleaning of duck sheds was practiced on a daily (70%, n = 14), weekly (5%, n = 1), and monthly (10%, n = 2) basis, and notably, 47% (n = 8) farms did not use disinfectant for cleaning purposes. Majority of the farmers (n = 17, 85%) washed hand with soap after contact with duck and handling of sick duck was avoided in 70% (n = 14) farms (see Additional file 5 showing variations in anthropogenic factors and environmental conditions).

**Fig. 4:**
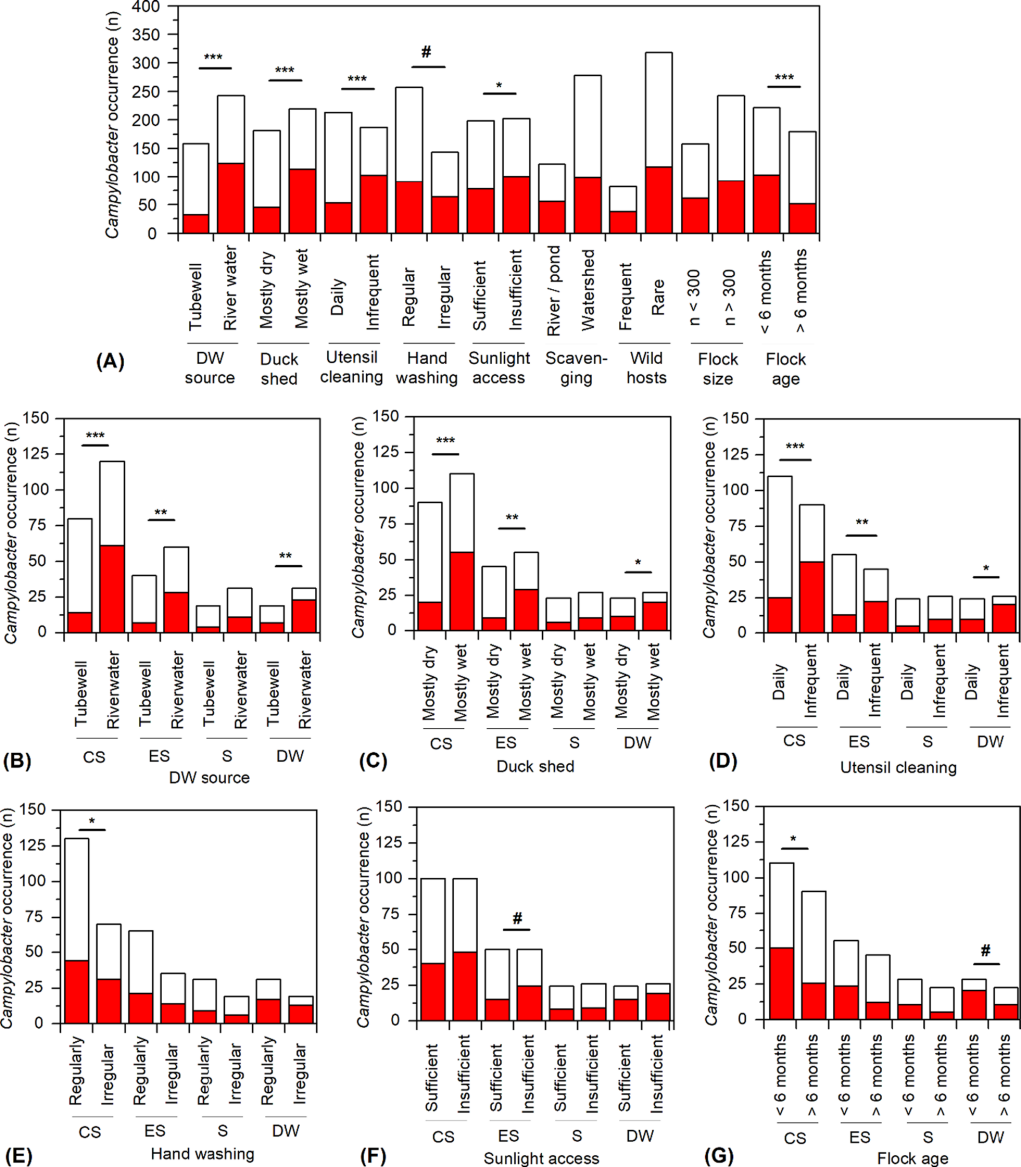
Occurrence of *Campylobacter* spp. in different samples of the selected duck farms in relation to the variable management practices. **A** Variation in the overall occurrence of *Campylobacter* spp. under various categories of farm management practices. **B**–**G** Influences of each of the potential driving factors on the occurrence of *Campylobacter* spp. in different sample types. CS, ES, S, and DW indicate the types of samples, i.e., cloacal swab, egg surface swab, the soil of the duck resting places, and drinking water, respectively. The filled and unfilled portions in the stacked bars of each column represent the number of samples (n) detected positive and negative, respectively, for the occurrence of *Campylobacter* spp. A Chi-square test was performed to determine the significant association between *Campylobacter* occurrence and each of the driving factors. ***, **, *, and # indicate p values of < 0.0005, < 0.005, < 0.05, and < 0.1, respectively. Details of statistical information are included in Additional file 6

Comparison with individual factors of farm management and environmental conditions with the isolation results of *Campylobacter* spp. revealed that source of drinking water, wet or dryness of duck shed, the practice of utensil cleaning, and flock age have profound impact (p < 0.0005) on the overall occurrence of *Campylobacter* spp. in duck farm samples (Fig. 4A). Among other factors, handwashing practice, and sunlight access to the resting place of the ducks were also observed to influence the bacterial preponderance but in less magnitude. Considering the influence of drinking water source, reduction in *Campylobacter* occurrence for using tube-well water in comparison to river water was observed to be highly significant (p < 0.0005) in cloacal swab samples and significant (p < 0.005) in egg surface swab and drinking water samples but insignificant for soil samples (Fig. 4B). As potential drivers, the wet condition of duck shed and irregularity in utensil cleaning were observed to induce *Campylobacter* occurrence in a similar fashion, highly significantly (p < 0.0005) in the cloacal swab, significantly (p < 0.005) in egg swab and with marginal significance (p < 0.05) for drinking water samples, but no such influence for soil samples (Fig. 4C and E). As a potential intervention, regular handwashing was found to be significant (p < 0.05) in reducing the risk of *Campylobacter* incidence in cloacal swab samples. Association with *Campylobacter* incidence of flock age 1–6 month and insufficient access to sunlight was found to be marginally significant for cloacal swab samples but not in other kinds of samples (Fig. 4F and G). Detail statistical information of the Chi-square tests observing the association of a particular factor of farm management with *Campylobacter* occurrence in different sample types are included in Additional file 6.

## Discussion

Being the major reservoirs of *Campylobacter* spp., poultry and livestock are mainly accountable for the continuing transmission of campylobacteriosis throughout the world. Among the agro-based enterprises, the poultry sector has been rapidly increasing, with an annual growth rate of 15–20% per annum, in densely-populated Bangladesh [38]. Duck farming is not only a promising livelihood alternative for the poverty-ridden communities in this country but also an important mean to enrich food security, particularly, in rural areas where plant-origin protein production is still challenging [39]. However, the rearing of ducks, an important reservoir for *Campylobacter* species, may impose an impending risk to public health. Recent studies have confirmed a close link between gastrointestinal disorders, caused by pathogenic microbes, including *Campylobacter* spp., with the pervasive incidence of malnutrition, particularly among children under 5 years, in Bangladesh and other developing countries [40]. Apart from the high prevalence of malnutrition, a rapid increase in disease incidences caused by MDR pathogens is the most challenging threat to public health in this developing nation with an agro-based economy. The large-scale use of antimicrobial agents, mostly in an imprudent manner, in the rapidly growing poultry sector is an important cause of MDR infections. The present study provides an insight into the occurrence, antimicrobial resistance traits, and potential risks of *Campylobacter* spp. associated with duck farming in Bangladesh, which could be credited as the first of its kind.

This survey has investigated the occurrence and its associated factors of *Campylobacter* in scavenging duck farms. The overall occurrence of *Campylobacter* spp. observed for the selected farms in this study is in congruence with a number of previous studies [41, 42]. However, in comparison to this study, there are reports of a higher incidence of *Campylobacter* occurrence in duck samples [43, 44]. The observed dominance of *C. coli* (ca. 61%) than *C. jejuni* (ca. 39%) in duck samples indicates a host-specific adaptive advantage of the former bacteria and in accordance with a number of previous investigations [43, 45]. There are also reports of a higher prevalence of *C. jejuni* than *C. coli* in duck samples [46–48]. Recent surveillance conducted in China has observed an overall prevalence of *Campylobacter* spp. in 33.5% of duck samples, similar to this study, but co-dominance of both *C. jejuni* and *C. coli* (ca. 49 and 47%, respectively) [49]. These variations could be attributable to the differences in climatic settings, sample types, and analytical methods [50].

In the poultry production and supply chain, a frequent occurrence of MDR strains of *Campylobacter* has been reported worldwide [51, 52]. Unwise applications of antimicrobial agents exert selective pressure on the exposed bacterial populations for the evolvement of resistant traits. The occurrence of MDR strains of *Campylobacter* spp. is primarily accredited to the large-scale and imprudent use of a variety of antimicrobials in the poultry and livestock sectors. In Bangladesh, antimicrobials are indiscriminately used by the poultry farmers, a vast majority of them following the biased instructions from the feed and chick traders, and their associated suppliers of antimicrobial agents [53]. Likewise, a large fraction of *C. jejuni* and *C. coli* populations in duck farms in this study has been observed as resistant to commonly used antimicrobials, e.g., amoxicillin, erythromycin, streptomycin, and tetracycline. Depending on variable geo-socio-climatic conditions, diverse patterns of resistance traits, e.g., a low frequency of erythromycin resistance but the high occurrence of resistance to ciprofloxacin and tetracycline among *Campylobacter* isolates from duck or poultry samples in Malaysia and Italy, have been noted [44, 54]. Considering the ‘One health’ perspective [53], the occurrence of MDR strains among ca. 37 and 42% of the *C. coli* and *C. jejuni* isolates, respectively, in the duck farm samples point out an alarming situation. Notably, health hazards from zoonotic infections by some of these MDR strains showing full or intermediate resistance to all major kinds of antimicrobials, including β-lactam, aminoglycoside, quinolone, and macrolide, is very problematic. A similar situation has been reported for the prevalent *Campylobacter* populations in chicken farms in the study region [11]. Although detected as comparatively less abundant than *C. coli*, the occurrence of MDR traits in equivalent or higher frequency in *C. jejuni* strains isolated from the duck farms (Table 2) could be related to the natural overwhelming predominance of the latter species in broiler meats and other poultry products and human campylobacteriosis [15, 40, 54]. Notwithstanding is the observed similarity in the percentage occurrence of different resistance traits between *C. jejuni and C. coli* strains (Fig. 2), indicating a salient role of horizontal gene transfer between these two species. MDR traits in *Campylobacter* strains are often linked to the presence of multiple resistant genes in large mobile elements (e.g., plasmids, Class I integron, conjugative transposon) in the bacterial genome [55]. Mutations or modulations in a number of genes regulating efflux pump systems, e.g., *gyrA* and *gyrB* of DNA gyrase, *parC*, and *parE* of topoisomerase IV, and the *cmeB* gene of the CmeABC multidrug efflux pump have been also shown to confer MDR traits, including resistance to fluoroquinolones, in *Campylobacter* spp. [56].

Occurrence in a portion of *C. jejuni* and *C. coli* strains of intermediate resistance for fluoroquinolones, azithromycin, and erythromycin can be attributable to the natural decay of their active components, and bacterial exposure to sub-lethal concentration of these compounds [11]. Antimicrobial residues, at sub-inhibitory concentrations, can trigger different biological effects, such as stress response, enhanced mutation rate, or genetic recombination events in bacterial populations, therefore, may have salient impacts to our gut flora [57]. Unfortunately, the residual content of commonly used antimicrobials, including fluoroquinolones, has been reported for the majority of broiler meats and livers in Bangladesh [11].

The transmission dynamics of antimicrobial-resistant bacteria to humans are related to the consumption of contaminated animal-source food, direct contact with animals, and exposure to contaminated environmental sources such as water and dust [58]. As noted in PE surveillance of this study, the large-scale use of antimicrobial agents in all the study farms, in combination with the extensive use of duck manure for aquaculture production may impose increased vulnerability to MDR pathogens among the local residents. Contamination with *Campylobacter* spp., including their MDR strains, of natural surface waters serving as a major source of drinking water for the reared ducks could be linked to the discarded duck feces. However, this could explain only partially the observed occurrence and diversity in antimicrobial resistance of *Campylobacter* spp., taking into account the reported use of antimicrobials at the study farms. For example, resistance to amoxicillin, erythromycin and streptomycin were observed for a vast majority of the isolated strains did not match with the antimicrobial agents applied most frequently (gentamicin, tetracycline, and fluoroquinolones) at the duck farms. Therefore, it could be assumed that allochthonous factors, including droppings from wild birds, and input of sewage materials from poultry farms nearby, play an integral role in the dynamics and resistance traits of *Aeromonas* spp. in natural surface waters, which are used without any treatment as drinking water in the majority of duck farms. The MDR traits prevalent among the *Aeromonas* strains in sources of drinking water may eventually correlate to the bacterial resistance patterns in the duck samples.

Analyses made in this study demonstrates that using of water from rivers or ponds rather than tube-well is significantly associated with the *Campylobacter* occurrence in not only DW but also in CS and ES samples, i.e., water pre-contaminated at natural reservoirs is a dominant factor linked to the bacterial preponderance in duck samples. This is in congruence with the relatively low die-off rate of *Campylobacter* in water [59]. The widespread occurrence of thermotolerant *Campylobacter* in natural surface waters is largely attributable to the frequent contamination of aquatic ecosystems with feces of the reservoir animals, including wild birds, and run-off from poultry farms or sewage [60]. Potable drinking waters can be also contaminated in the farm environment due to a lack of regular hygienic practices, particularly, utensil cleaning, and significantly influence *Campylobacter* incidences in CS and ES samples (Fig. 4). Insufficient disinfection treatment of drinking water has been recognized as a significant driver of *Campylobacter* incidence in the production and supply chain of broiler chicken [11, 26]. Based on the results of this study, decontaminating drinking water before serving to the reared ducks, and proper treatment of duck waste products before discarding them to adjacent aquatic ecosystems could be distinguished as important interventions to reduce the risk of MDR infections caused by *Campylobacter* spp. derived from the duck farms.

Variation in the monthly occurrence of *Campylobacter* spp. in the farm samples of this study could be linked to the variable amplitude of climatic factors. Particularly, a significant role of increased temperature and humidity in stimulating the bacterial occurrence was discernible. *Campylobacter* colonization in the intestine and caeca of chicken is reported to fluctuate with seasonal variation in temperature [12]. A significant increase in the incidences of *Campylobacter* infection during the summer months has been reported for many countries, particularly in the temperate region [16]. However, the observed negative influence of increased sunshine hour on *Campylobacter* occurrence in duck farm samples could be related to retardation of the bacterial populations with intensified ultraviolet radiation [61]. On the other hand, a correlation between high humidity and increased *Campylobacter* occurrence observed for the duck farm samples in this study is in agreement with results from clinical surveillance and broiler farms [14, 62]. Increased precipitation and relative humidity can aid to prolonged persistence of *Campylobacter* spp. in the environment since the bacteria is sensitive to dry conditions [14]. A positive influence of increased rainfall on *Campylobacter* incidence in the farm samples was also evident from this study, although a drastic decrease in the bacterial occurrence during July (Fig. 2) could be attributable to the dilution impact of high precipitation events. Elucidated influences of multiple climatic factors on *Campylobacter* occurrence may not always be discernible since the inferences are based on short-term observations. Previous investigations at different geo-socio-climatic settings noted that the bacterial infection dynamics could partially correlate to the climatic factors [63, 64]. Nonetheless, the estimated regulation of each of the climatic drivers provides a basis to predict the bacterial dynamics in duck farms.

In densely-populated countries like Bangladesh, proper implementation of management strategies to minimize the risks of *Campylobacter* and other zoonotic infections from the growing poultry industries are crucial. Concerning environmental management, remarkable is the observed significance between *Campylobacter* occurrence and persistence of wet condition, and insufficient sunlight in duck sheds, which also support the inferences made for climatic influences on the bacterial dynamics. As discussed above, using untreated natural surface water as drinking water, infrequent cleaning of utensils, and inadequate application of disinfectants are potential risk factors of *Campylobacter* occurrence in duck farms. Farm managers should also think of intervention strategies considering the age-specific variable susceptibility of ducks to *Campylobacter* infection. Chicken flocks aged > 30 days are considered more susceptible to *Campylobacter* infection than younger chicks at broiler farms in the study region [26]. Interestingly, the results of this study indicate that older ducks above 6 months are a less potent reservoir of the bacteria. Although not evident from the observations made in this study, a larger flock size (> 1500 birds) of poultry animals could be occasionally important determinant of higher *Campylobacter* occurrence [26].

Adoption of proper management practices for not only duck rearing but also maintaining a healthy environment is largely credited to the enriched knowledge and educational status of the farmers. Sociodemographic information obtained in this study showed that most of the farmers lacked training on duck rearing methods and many of them discontinued formal education (Additional file 4). These lacking in education and learning-based improved knowledge could have indulged inadequate management practices among the majority farmers favoring the enhanced incidence of *Campylobacter* occurrence in the duck farms. A recent surveillance study in Bangladesh has also reported that persons having a long term (10 years or more) experience in poultry farming and graduate-level education, are more likely to use antimicrobial agents in a wise manner [65]. Unfortunately, the majority of the duck rearers in this study had the farming experience of fewer than 5 years and none had completed graduate-level education. Imprudent application of antimicrobial agents in the study farms could be also attributable to the lack of animal health care following guidelines of the registered veterinarians, as reported by a vast majority (80%) of the duck rearers, Moreover, these farmers did not receive any training on biosecurity measures that could play a significant role in limiting their large-scale application of antimicrobial agents, and disposal of fecal waste from the reared ducks into natural water reservoirs and adopting other environmental interventions or protective measures to minimize the risk of occurrence, and dissemination of MDR strains of *Campylobacter* spp.

An important recent strategy to ensure food safety is the “farm to fork” approach including a comprehensive understanding of the hazards and control points at all stages of the food production and supply chain. Therefore, proper tackling of *Campylobacter* infections would require holistic understanding of the interactive linkages of disease epidemiology with bacterial transmission dynamics, associated environmental drivers and anthropogenic practices at poultry farms. Inferences made in this study on potential sources and risk factors of *Campylobacter* occurrence would lead to formulating an integrated management approach to prevent or control *Campylobacter* infection from ducks reared in homestead farms. However, analysis of larger samples through a logistic regression model could signify the correlation between the risk factors and *Campylobacter* occurrence. In conjunction, case–control studies comparing the occurrence and genetic traits of potentially harmful *Campylobacter* spp. in ducks and among the infected human populations would provide more insights into the extent of the associated socio-environmental determinants.

## Conclusion

Results obtained in this study show that duck farms are a potential source of *Campylobacter* spp. and may contribute to the incidences of human campylobacteriosis in Bangladesh. The occurrence of *C. coli* and *C. jejuni* in duck farms is influenced by a combined interaction of environmental and anthropogenic factors. Among the climatic variables, temperature and humidity are the major drivers of *Campylobacter* dynamics. Untreated natural surface water, if used as drinking water for the ducks contributes significantly to the increased occurrence of *Campylobacter* spp. An important concern is that a large number of *C. jejuni* and *C. coli* strains have become resistant to multiple antimicrobials. Anthropogenic interventions including regular cleaning of utensils, frequent hand washing, provision of sufficient ventilation, and sunlight access are inferred as potentially preventive to *Campylobacter* infections. The study also suggests an inherent need for awareness building among the farmers to promote adequate practices regarding hygiene, water treatment, waste disposal, and adept use of antimicrobial agents to reduce the spread of MDR *Campylobacter* and health hazards associated with duck farming. Therefore, proper tackling of *Campylobacter* infections in humans would require not only detailed information on disease epidemiology but also a holistic understanding on how the bacterial transmission dynamics are influenced by the combined impacts of climatic, anthropogenic, environmental factors. Future research should focus on horizontal transfer of pathogenic traits among *Campylobacter* strains isolated from ducks, poultry, wild avian fauna, and human populations and explore comparative genome analysis tools at the environment-human-animal interfaces.

## Supplementary Information

**Additional file 1. **Questionnaire for assessment of sociodemographic information, farm management and hygienic practices in semi-scavenging duck farms at Mymensingh district of Bangladesh.

**Additional file 2.** Representative gel images with PCR-amplicons detecting *Campylobacter* genus by 16S rRNA gene-based PCR, and the predominant species, *C. jejuni* and *C. coli*, by *hipO* gene-, and *cdtC* gene-based PCR assays.

**Additional file 3. **Variation in monthly isolation rate of *Campylobacter* spp. in different samples collected from the selected duck farms.

**Additional file 4. **Sociodemographic status of duck rearers (N = 57) at selected duck farms in Bangladesh.

**Additional file 5.** Status of farm management operations, hygiene and sanitation practices at selected duck farms (n = 20) in Bangladesh.

**Additional file 6. **Probable role of anthropogenic and environmental factors on the occurrence of *Campylobacter* spp.

## Data Availability

All datasets analyzed during this research are included in this published article [and its Additional files]. Detail information on the datasets and materials used in this study are available from the corresponding author on reasonable request. Confidentiality of data is maintained anonymously.

## Abbreviations

BBA: Blood agar base no.2
CI: Confidence interval
FGD: Focus group discussion
IQR: Interquartile range
MDR: Multi drug resistance
PCR: Polymerase chain reaction
PE: Participatory epidemiology
CS: Cloacal swab
ES: Egg surface swab
S: Soil of the duck resting places
DW: Drinking water
F: Feed
AMX: Amoxicillin
AZM: Azithromycin
CIP: Ciprofloxacin
ERY: Erythromycin
GEN: Gentamycin
NOR: Norfloxacin
STR: Streptomycin
TET: Tetracycline
