# Supplementary material for: Occurrence and multidrug resistance of Campylobacter spp. at duck farms and associated environmental and anthropogenic risk factors in Bangladesh
Source: BMC Infect Dis. 2021 Nov 7;21:1139. doi: 10.1186/s12879-021-06834-w (PMC8574054; doi:10.1186/s12879-021-06834-w)
Supplement: Supplementary file 1 — Additional file 1. Questionnaire for assessment of sociodemographic information, farm management and hygienic practices in semi-scavenging duck farms at Mymensingh district of Bangladesh. [file 12879_2021_6834_MOESM1_ESM.docx]

**Additional file 1.** **Questionnaire for assessment of sociodemographic information, farm management and hygienic practices in semi-scavenging duck farms at Mymensingh district of Bangladesh**

| Questionnaire for assessment of sociodemographic information, farm management along with cleaning and personal hygienic practices through semi-structure interview (SSI)/focus group discussion (FGD)/visualization/transect work in semiscavenging duck farms of Mymensingh district of Bangladesh | | | | | | | | | | | | | | | | | | | | | | | | | | | | | | | | | | | | | | | | | | | | | | | | | |
| --- | --- | --- | --- | --- | --- | --- | --- | --- | --- | --- | --- | --- | --- | --- | --- | --- | --- | --- | --- | --- | --- | --- | --- | --- | --- | --- | --- | --- | --- | --- | --- | --- | --- | --- | --- | --- | --- | --- | --- | --- | --- | --- | --- | --- | --- | --- | --- | --- | --- |
| Date of Interview: | | | | | | | | | ………/………/………… | | | | | | | | | | | | | | | | | | | | | |  | | | | | | | ID No.: | | | | | | | | | | | |
| **A. Socio-demographic information** | | | | | | | | | | | | | | | | | | | | | | | | | | | | | | | | | | | | | | | | | | | | | | | | | |
| 1. GPS Coordinates: | | | | | | | | | a) Northing | | | | | | | | | | | |  | | | | | | | | | | b) Easting: | | | | | | | | | | | | | | | | | | |
| 2. Name of the interviewee: | | | | | | | | |  | | | | | | | | | | | | Mobile no. | | | | | | | | | |  | | | | | | | | | | | | | | | | | | |
| 3. Farm location: | | | |  | | | | | | | | | | | | | | | | | | | | | | | | | | | Upazila: | | | | | | | | |  | | | | | | | | | |
| 4. Profession: | | | | | | | | | | | | | | | | | | | | | | | | | | | | | | | | | | | | | | | | | | | | | | | | | |
| Farmer (Owner) | | | |  | | | | | Son (Support Staff) | | | | | | | | | | | |  | | | | | | | | | | Wife (Support staff) | | | | | | | | | | | | | | | | | | |
|  | | | |  | | | | | Hired employee (Support staff) | | | | | | | | | | | | | | | | | | | | | | | | | | | | | | | | | | | | | | | | |
| 5. Sex | | | | | | | | | Male | | | |  | | | | | | | | Female | | | | | | | | | | | | | | | | |  | | | | | | | | | | | |
| 6.Educational status | | | | | | | | | | | | | | | | | | | | | | | | | | | | | | | | | | | | | | | | | | | | | | | | | |
|  | No formal education | | | | | | | |  | | | | SSC | | | | | | | |  | | | | | | | | | | HSC | | | | | | | | | | | | | | | | | | |
|  | Class (VI-X) | | | | | | | |  | | | |  | | | | | | | |  | | | | | | | | | |  | | | | | | |  | | | | | | | | | | | |
| 7. Training on duck rearing: Yes/No | | | | | | | | | | | | | | | | | | | | | | | | | | | | | | | | | | | | | | | | | | | | | | | | | |
| 8. Experience in duck rearing (years) | | | | | | | | | | | | | | | | | |  | | | 1 to 5 | | | | | | | | | |  | | | 5 to 10 | | | | | | | | | |  | | >10 | | | |
| **B. Farm management, cleaning and sanitation practices and personal hygiene** | | | | | | | | | | | | | | | | | | | | | | | | | | | | | | | | | | | | | | | | | | | | | | | | | |
| 1. Flock size(number of ducks in each farm) | | | | | | | | | | | | | | | | |  | | | <300 | | | | | | | | |  | | 300-750 | | | | | | | |  | | | | 750-1500 | | | | | | |
| 2. Age of the birds(months) | | | | | | | | | | | | | | | | | | |  | | 1 to 5 | | | | | | | | | |  | | | | | 10 to 15 | | | | | |  | | | | | | >15 | |
| 3. Breed of the birds | | | | | | | |  | Choruy & Khaki Chambel | | | | | | | | | | | | | | |  | | | | | | Choruy | | | | | | | |  | | Khaki Chambel | | | | | | | | | |
| 4. Scavenging side | | | | | | | |  | Watershed and paddy field | | | | | | | | | | | | | | |  | | | | | | River and paddy field | | | | | | | | | | | | | | | | | | | |
|  |  |  |  |  |  |  |  |  | Pond and paddy field | | | | | | | | | | | | | | | | | | | | | | | | | | | | | | | | | | | | | | | | |
| 5. Feed | |  | | Paddy | | | | |  | | Paddy, wheat bran and commercial feed | | | | | | | | | | | | | | | | | | | | | | | | | | | | | | | | | | | | | | |
|  |  |  | | Paddy plus commercial feed | | | | | | | | | | | | | | | | |  | | | Rice police and commercial feed | | | | | | | | | | | | | | | | | | | | | | | | | |
| 6. Source of water | | | | | |  | | | River/pond | | | | | | | | | | | |  | | | Deep tube well | | | | | | | | | | | | | | | | | | | | | | | | | |
| 7. Floor type | | |  | | Concrete/brick | | | | | | | | | | | | | | | |  | | | | Wood/bamboo | | | | | | | | | | | | | | | | | | | | | | | | |
| 8. Floor condition | | | | | | | | |  | | | | Wet | | | | | | | |  | | | | | | | | | | Dry | | | | | | | | | | | | | | | | | | |
| 9. Sunlight and ventilation facilities | | | | | | | | | | | |  | | | | Yes | | | | | | | | | |  | | | | | | No | | | | | | | | | | | | | | | | | |
| 10. Cleaning of floor | | | | | | |  | | Never clean | | | | | |  | | 1x daily | | | | | | | | | |  | | | | 1x weekly | | | | | | | | | |  | | | | 1x Monthly | | | | |
| 11. Disinfectants(lime, potassium per manganite , Savlon) for cleaning of floor | | | | | | | | | | | | | | | | | | | | | | | | | | | | | | | | | | | | | | | | | | | | | | | | | |
|  | Yes | | | | | | | |  | | | | No | | | | | | | | | | | | | | | | | | | | | | | | | | | | | | | | | | | | |
| 12.Feeder and drinker cleaning | | | | | | | | | | | | |  | | | Yes | | | | | | |  | | | | | | | | No | | | | | | | | | | | | | | | | | | |
| 13. Veterinary health care facilities (like use of antibiotics and other drugs, vaccination) ensured by veterinarian’s advice | | | | | | | | | | | | | | | | | | | | | | | | | | | | | | | | | | | | | | | | | | | | | | | | | |
|  | Yes | | | | | | | |  | | | | No | | | | | | | | | | | | | | | | | | | | | | | | | | | | | | | | | | | | |
| 14. Therapeutics use of antibiotics mostly | | | | | | | | | | | | | | | | | | | | | | | | | | | | | | | | | | | | | | | | | | | | | | | | | |
|  | Gentamycin | | | | | | | |  | | | | Oxytetracycline | | | | | | | |  | | | | | | | | | | Enrofloxacin | | | | | | | | | | | | | | | | | | |
|  | Others: | | | | | | | | | | | | | | | | | | | | | | | | | | | | | | | | | | | | | | | | | | | | | | | | |
| 15.Use vaccine(duck plague and duck cholera) as control of duck diseases | | | | | | | | | | | | | | | | | | | | | | | | | | | | | | | | | | | | | | | | | | | | | | | | | |
|  | Yes | | | | | | | |  | | | | No | | | | | | | | | | | | | | | | | | | | | | | | | | | | | | | | | | | | |
| 16.Interface wild animal/bird-ducks | | | | | | | | | | | | |  | | Yes | | | | | | | | | | | | | | | |  | | | | | | | No | | | | | | | | | | | |
| 17.Duck manure use purpose | | | | | | | | | |  | | | | Fertilizer in agriculture field | | | | | | | | | | | | | | | | | | |  | | | | Fish feed in ponds | | | | | | | | | | | | |
| 18. Washing of hand with soap after contact with ducks | | | | | | | | | | | | | | | | | | | | | | | | | | | | | | |  | | | | | | | Yes | | | | | | | | |  | | No |
| 19. Occurrence of gastroenteritis/diarrhea | | | | | | | | | | | | | | | |  | No | | | | |  | | | | | | Yes, Frequency_ _ _ _ _ _ _ _ _ | | | | | | | | | | | | | | | | | | | | | |
| 20. Handling of ducks during physical sickness like fever, diarrhea | | | | | | | | | | | | | | | | | | | | | | | | | | | | | | | | | | |  | | | Yes | | | | | | | | |  | | No |
| 21. Maintain Personnel Hygiene Properly? | | | | | | | | | | | | | | | | | | | | | | | | | | | | | | | | | | |  | | | Yes | | | | | | | | |  | | No |
| **C. Other comment(s) /observation(s) on risk factors (if any):** | | | | | | | | | | | | | | | | | | | | | | | | | | | | | | | | | | | | | | | | | | | | | | | | | |
| **Verification of data**  Collected by: Verified by:  ____________________________________ ________________________________  Name, Signature & Date Name, Signature & Date | | | | | | | | | | | | | | | | | | | | | | | | | | | | | | | | | | | | | | | | | | | | | | | | | |
