# Supplementary material for: Occurrence and multidrug resistance of Campylobacter spp. at duck farms and associated environmental and anthropogenic risk factors in Bangladesh
Source: BMC Infect Dis. 2021 Nov 7;21:1139. doi: 10.1186/s12879-021-06834-w (PMC8574054; doi:10.1186/s12879-021-06834-w)
Supplement: Supplementary file 2 — Additional file 2. Representative gel images with PCR-amplicons detecting Campylobacter genus by 16S rRNA gene-based PCR, and the predominant species, C. jejuni and C. coli, by hipO gene-, and cdtC gene-based PCR assays. [file 12879_2021_6834_MOESM2_ESM.docx]

**Additional file 2. Representative gel images with PCR-amplicons detecting *Campylobacter* genus by 16S rRNA gene-based PCR, and the predominant species, *C. jejuni* and *C. coli*, by *hipO* gene-, and *cdtC* gene-based PCR assays**


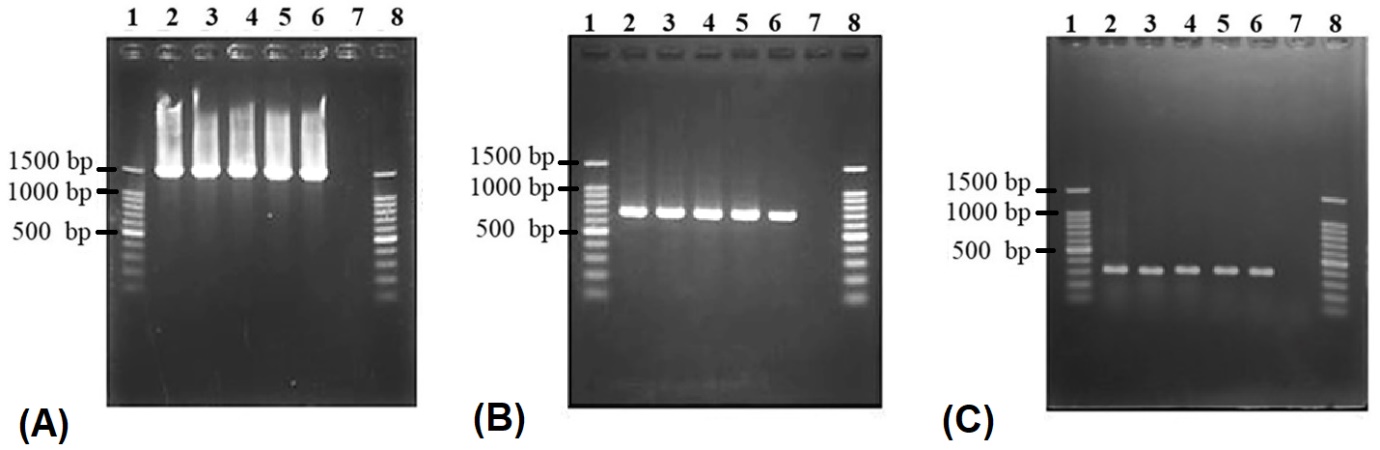


Figures showing gel images with amplified products of (A) 16S rRNA gene-based PCR to identify *Campylobacter* genus, (B) *hipO* gene-based PCR assay to identify *C. jejuni*, and (C) *cdtC* gene-based multiplex PCR assay to identify *C. coli*. The PCRs were run individually and a 5 µl portion of each of the PCR amplicons were electrophoresed in 2 % agarose gel. Gel images were captured after staining and destaining of the electrophresed products following an identical protocol. In all figures, lanes: 1 and 8, 100 bp DNA ladder (Promega, USA); lane 2, amplified PCR product of positive control (genomic DNA of *C. jejuni* ATCC 33560 in A and B; and *C. coli* ATCC 33559 in C); lanes 3-6, PCR amplified products of the genomic DNA of representative *Campylobacter* spp. (*C. coli* in A and C; and *C. jejuni* in B) isolated from the duck farm samples; lane 7, negative control (sterile deionized water).
