# Supplementary material for: Occurrence and multidrug resistance of Campylobacter spp. at duck farms and associated environmental and anthropogenic risk factors in Bangladesh
Source: BMC Infect Dis. 2021 Nov 7;21:1139. doi: 10.1186/s12879-021-06834-w (PMC8574054; doi:10.1186/s12879-021-06834-w)
Supplement: Supplementary file 3 — Additional file 3. Variation in monthly isolation rate of Campylobacter spp. in different samples collected from the selected duck farms. [file 12879_2021_6834_MOESM3_ESM.docx]

**Additional file 3. Variation in monthly isolation rate of *Campylobacter* spp. in different samples collected from the selected duck farms**

| **Month** | **CS** | **ES** | **S** | **W** | **Total** |
| --- | --- | --- | --- | --- | --- |
| April | 17.5 % | 20.0 % | 8.33 % | 16.67 % | 17.07 % |
|  | (n = 40) | (n = 20) | (n = 9) | (n = 9) | (n = 78) |
| May | 40.0 % | 36.0 % | 30.0 % | 53.33 % | 38.46 % |
|  | (n = 50) | (n = 25) | (n = 12) | (n = 12) | (n = 99) |
| June | 50.0 % | 48.0 % | 33.33 % | 68.33 % | 49.06 % |
|  | (n = 50) | (n = 25) | (n = 14) | (n = 14) | (n = 103) |
| July | 20.0 % | 20.0 % | 16.67 % | 33.33 % | 25.0 % |
|  | (n = 30) | (n = 15) | (n = 8) | (n = 8) | (n = 61) |
| August | 56.67% | 46.67 % | 44.44 % | 100.0 % | 54.84 % |
|  | (n = 30) | (n = 15) | (n = 7) | (n = 7) | (n = 7) |
| Overall | 37.5 % | 35.0 % | 30.0 % | 60.0 % | 38.75 % |
|  | (n = 200) | (n = 100) | (n = 50) | (n = 50) | (n = 400) |
