# Supplementary material for: Occurrence and multidrug resistance of Campylobacter spp. at duck farms and associated environmental and anthropogenic risk factors in Bangladesh
Source: BMC Infect Dis. 2021 Nov 7;21:1139. doi: 10.1186/s12879-021-06834-w (PMC8574054; doi:10.1186/s12879-021-06834-w)
Supplement: Supplementary file 4 — Additional file 4. Sociodemographic status of duck rearers (N = 57) at selected duck farms in Bangladesh. [file 12879_2021_6834_MOESM4_ESM.docx]

**Additional file 4. Sociodemographic status of duck rearers (N=57) at selected duck farms in Bangladesh**

| Factor | Category | Frequency |  |
| --- | --- | --- | --- |
|  |  | Number | % |
| Profession | Farmer (owner) | 20 | 35.10 |
|  | Family member: wife(spouse) | 17 | 29.82 |
|  | Family member: son | 10 | 17.54 |
|  | Hired employees | 10 | 17.54 |
| Sex | Male | 37 | 64.90 |
|  | Female | 20 | 35.10 |
| Education status | No formal education | 14 | 24.56 |
|  | Primary level (Class I-V) | 13 | 22.80 |
|  | Secondary level (Class VI-X) | 13 | 22.80 |
|  | Secondary School Certificate(SSC) | 11 | 19.30 |
|  | Higher Secondary Certificate(HSC) | 6 | 10.54 |
| Training on duck rearing and biosecurity | Yes | 3 | 5.26 |
|  | No | 54 | 94.74 |
| Experience in duck | 1 to 5 | 37 | 64.91 |
| rearing (years) | 5 to 10 | 11 | 19.30 |
|  | >10 | 9 | 15.79 |

SSC= Secondary School Certificate, HSC= Higher Secondary School Certificate
