# Supplementary material for: Occurrence and multidrug resistance of Campylobacter spp. at duck farms and associated environmental and anthropogenic risk factors in Bangladesh
Source: BMC Infect Dis. 2021 Nov 7;21:1139. doi: 10.1186/s12879-021-06834-w (PMC8574054; doi:10.1186/s12879-021-06834-w)
Supplement: Supplementary file 5 — Additional file 5. Status of farm management operations, hygiene and sanitation practices at selected duck farms (n = 20) in Bangladesh. [file 12879_2021_6834_MOESM5_ESM.docx]

**Additional file 5.** Status of farm management operations, hygiene and sanitation practices at selected duck farms (n = 20) in Bangladesh

| **Factor** | **Category** | **Frequency number (n)** | **%** |
| --- | --- | --- | --- |
| Breed of ducks | Choruy | 2 | 10 |
|  | Khaki Chambal | 16 | 80 |
|  | Choruy & Khaki Chambal | 2 | 10 |
| Flock size (number of ducks) | <300 (small) | 8 | 40 |
|  | 300-750 (median) | 6 | 30 |
|  | 750-1500 (Large) | 6 | 30 |
| Age of the ducks (months) | 1 to 5 | 11 | 55 |
|  | 10 to 15 | 8 | 40 |
|  | >15 | 1 | 5 |
| Scavenging area | Watershed and paddy field | 14 | 70 |
|  | River and paddy field | 4 | 20 |
|  | Pond and paddy field | 2 | 10 |
| Source of drinking water | River/pond | 12 | 60 |
|  | Deep tube well | 8 | 40 |
| Floor condition | Wet | 11 | 55 |
|  | Dry | 9 | 45 |
| Sun light and ventilation facilities | No | 10 | 50 |
|  | Yes | 10 | 10 |
| Cleaning the floor of duck shed | Never clean | 3 | 15 |
|  | 1X daily | 14 | 70 |
|  | 1X weekly | 1 | 5 |
|  | 1X monthly | 2 | 10 |
| Disinfectants (lime, potassium per manganite, savlon) for floor cleaning (n=17) | No (use only water) | 8 | 47 |
|  | Yes | 9 | 53 |
| Feeder and drinker cleaning | Yes | 12 | 60 |
|  | No | 8 | 40 |
| Veterinary health care (use of antibiotics, other drugs and vaccine) following the advice of veterinarian | Yes | 4 | 20 |
|  | No | 17 | 80 |
| Therapeutics use of antibiotics mostly | Gentamicin | 10 | 50 |
|  | Oxytetracycline | 4 | 20 |
|  | Enrofloxacin | 6 | 30 |
| Use of vaccine (duck plague and duck cholera) | Yes | 12 | 60 |
|  | No | 8 | 40 |
| Interface wild animal/bird-ducks | Yes | 4 | 20 |
|  | No | 16 | 80 |
| Use of duck manure | Fertilizer in agriculture field | 3 | 15 |
|  | Fish feed in ponds | 17 | 85 |
| Washing of hand with soap after contact with ducks | Yes | 17 | 85 |
|  | No | 3 | 15 |
| Handling of ducks during physical sickness like fever and diarrhoea | Yes | 6 | 30 |
|  | No | 14 | 70 |
