# Supplementary material for: Occurrence and multidrug resistance of Campylobacter spp. at duck farms and associated environmental and anthropogenic risk factors in Bangladesh
Source: BMC Infect Dis. 2021 Nov 7;21:1139. doi: 10.1186/s12879-021-06834-w (PMC8574054; doi:10.1186/s12879-021-06834-w)
Supplement: Supplementary file 6 — Additional file 6. Probable role of anthropogenic and environmental factors on the occurrence of Campylobacter spp. [file 12879_2021_6834_MOESM6_ESM.docx]

**Additional file 6. Probable role of anthropogenic and environmental factors on the occurrence of *Campylobacter* spp.**

| **Category** | **Variables** | **Occurrence in CS** | | | **Occurrence in ES** | | | **Occurrence in S** | | | **Occurrence in DW** | | | **Overall occurrence** | | |  |
| --- | --- | --- | --- | --- | --- | --- | --- | --- | --- | --- | --- | --- | --- | --- | --- | --- | --- |
|  |  | **Pos** | **Neg** | ***X*^2^ (2,**  **N =200)** | **Pos** | **Neg** | ***X*^2^ (2,**  **N =100)** | **Pos** | **Neg** | ***X*^2^ (2,**  **N = 50)** | **Pos** | **Neg** | ***X*^2^ (2,**  **N = 50)** | **Pos** | **Neg** | ***X*^2^ (2,**  **N = 400)** | |
| DW | Tube-well | 14 | 66 | 22.7556, | 7 | 33 | 8.9744, | 4 | 15 | 1.1682, | 7 | 12 | 6.8478, | 32 | 126 | 37.646, | |
| source | River | 61 | 59 | p <0.00001 | 28 | 32 | p =0.0027 | 11 | 20 | p =0.27976 | 23 | 8 | p =0.00888 | 123 | 119 | p <0.0001 | |
| Utensil | Daily | 25 | 85 | 22.7609, | 13 | 42 | 6.9375, | 5 | 19 | 1.8468, | 10 | 14 | 6.4640, | 53 | 160 | 36.9155, | |
| cleaning | Infrequent | 50 | 40 | p <0.00001 | 22 | 23 | p =0.0084 | 10 | 16 | p =0.17420 | 20 | 6 | p =0.0110 | 102 | 85 | p <0.0001 | |
| Duck | Mostly dry | 20 | 70 | 16.2963, | 9 | 36 | 11.2517, | 6 | 17 | 0.3106, | 10 | 13 | 4.8443, | 45 | 136 | 29.6424, | |
| shed | Mostly wet | 55 | 55 | p =0.00005 | 29 | 26 | p =0.0008 | 9 | 18 | p = 0.52878 | 20 | 7 | p =0.02774 | 113 | 106 | p <0.0001 | |
| Flock | N <300 | 31 | 49 | 0.0889, | 15 | 25 | 0.1832, | 5 | 14 | 0.1981, | 11 | 8 | 0.0566, | 62 | 96 | 0.0267, | |
| size | N >300 | 44 | 76 | p =0.76559 | 20 | 40 | p =0.6686 | 10 | 21 | p =0.65627 | 19 | 12 | p =0.81196 | 93 | 149 | p = 0.87075 | |
| Flock | < 6 months | 50 | 60 | 6.5993, | 23 | 32 | 2.4975, | 10 | 18 | 0.9895, | 20 | 8 | 3.4632, | 103 | 118 | 12.8428, | |
| age | > 6 months | 25 | 65 | p =0.0102 | 12 | 33 | p =0.1140 | 5 | 17 | p =0.31986 | 10 | 12 | p =0.0627 | 52 | 127 | p = 0.00034 | |
| Hand | Regularly | 44 | 86 | 4.3269, | 21 | 44 | 0.5917, | 9 | 22 | 0.0364, | 17 | 14 | 0.9055, | 91 | 166 | 3.3818, | |
| washing | Irregular | 31 | 39 | p =0.03751 | 14 | 21 | p =0.4418 | 6 | 13 | p =0.84870 | 13 | 6 | p =0.3413 | 64 | 79 | p =0.06592 | |
| Sunlight | Sufficient | 40 | 60 | 1.2987, | 15 | 35 | 3.4048, | 8 | 16 | 0.0091, | 15 | 9 | 0.6416, | 78 | 120 | 4.139, | |
| access | Insufficient | 48 | 52 | p =0.2545 | 24 | 26 | p =0.0650 | 9 | 17 | p =0.92380 | 19 | 7 | p = 0.4231 | 100 | 102 | p =0.0419 | |
| Wild | Frequent | 18 | 22 | 1.2000, | 9 | 11 | 0.2364, | 3 | 8 | 0.05, | 8 | 3 | 0.9518, | 38 | 44 | 2.5045, | |
| hosts | Rare | 57 | 103 | p =0.27332 | 26 | 54 | p =0.62685 | 12 | 27 | p =0.82315 | 22 | 17 | p = 0.3293 | 117 | 201 | p =0.11352 | |
| Scaven- | River /pond | 27 | 33 | 2.0571, | 12 | 18 | 0.471, | 6 | 10 | 0.6373, | 11 | 5 | 0.7506, | 56 | 66 | 3.7828, | |
| ging | Watershed | 48 | 92 | p =0.15149 | 23 | 47 | p =0.49255 | 9 | 25 | p =0.42726 | 19 | 15 | p =0.38628 | 99 | 179 | p =0.05178 | |
